# A Randomized Controlled Trial of Chuanhutongfeng Mixture for the Treatment of Chronic Gouty Arthritis by Regulating miRNAs

**DOI:** 10.1155/2019/5917269

**Published:** 2019-02-03

**Authors:** Yao Wang, Liping Dong, Peng Liu, Ying Chen, Shaodan Jia, Yangang Wang

**Affiliations:** ^1^Department of Endocrinology, The Affiliated Municipal Hospital of Qingdao University, Qingdao 266011, China; ^2^Department of Endocrinology, Laiwu City People's Hospital, Laiwu 271100, China; ^3^Department of Endocrinology, The Affiliated Hospital of Qingdao University, Qingdao 266003, China; ^4^Department of Healthcare, The Affiliated Municipal Hospital of Qingdao University, Qingdao 266011, China

## Abstract

**Background:**

We investigated whether Chuanhutongfeng mixture has actions on chronic gouty arthritis (CGA) by regulating miRNAs.

**Methods:**

A total of 255 patients with CGA and 30 controls were enrolled. miRNA expression profiles and cluster analysis were preformed; RT-qPCR was used to detect miRNAs associated with CGA. Patients were allocated into Chuanhutongfeng mixture, allopurinol (positive control), and control (etoricoxib) groups. Expression of plasma miRNAs was measured before and after treatments; expression of chemokine 2 (CCL2) and interleukin 8 (CXCL8) was determined by ELISA.

**Results:**

48 miRNAs were differentially expressed and compared to controls. 36 miRNAs expression levels were > 1.5 times and 12 miRNAs < 1.5 times compared to the controls. miR-339-5p, miR-486-5p, and miR-361-5p levels in patients with CGA were lower than in controls (*P *< 0.05). This trial showed that the Chuanhutongfeng mixture and allopurinol groups had upregulated the expressions of miR-486-5, miR-339-5p, and miR-361-5p and decreased levels of CCL2 and CXCL8 proteins. After 8 weeks of treatment, Chuanhutongfeng mixture decreased serum uric acid levels more than allopurinol (*P *< 0.05) and reduced levels of CCL2 and CXCL8 protein significantly more than in the allopurinol and control groups.

**Conclusions:**

The therapeutic actions of Chuanhutongfeng mixture inhibit the expression of proteins CCL2 and CXCL8 in plasma and upregulated the expressions of three miRNAs (miR-486-5p, miR-339-5p, and miR-361-5p).

## 1. Introduction

 Gout is caused by monosodium urate monohydrate (MSU) depositions in synovial fluid and other tissues and characterized by bursal or peripheral joint swelling, tenderness, and pain, with MSU crystals in a symptomatic joint, bursa or tophus [1]. Interventions include nonpharmacological measures such as treatment of comorbidities, decrease in calorie intake, and alcohol consumption as well as avoidance of high-fructose corn syrup and alcoholic and nonalcoholic beer, heavy meals, and excessive intake of meat and seafood. The major target of pharmacological interventions is hyperuricemia (HU) through a reduction of serum uric acid (SUA) concentrations [2, 3]. These urate lowering therapies (ULTs) include the xanthine oxidase inhibitors (allopurinol, febuxostat), medications that increase urate excretion (probenecid, benzbromarone, and lesinurad), and recombinant uricase (pegloticase), which breaks down urate to water-soluble allantoin. However, all of the ULTs have certain contraindications [4, 5] and their prescriptions must be individually evaluated. Recommendations for management of gout flares include nonsteroidal anti-inflammatory drugs (NSAIDs), prednisolone, intra-articular injection of corticosteroids, and colchicine [3], the later drug also being used for anti-inflammatory prophylaxis [6]. The pathogenesis of gouty arthritis has also been correlated with IL-1*β* [7] via TNF-*α* [8], and NLRP3 inflammasome activation in macrophages [9] and chemokines [10] and an IL-1*β* antibody has been shown to provide superior inflammation prophylaxis than colchicine in patients with gout [11]. MicroRNAs are defined as noncoding RNAs consisting of 19 to 24 nucleotides. miRNAs achieve negative regulation by binding specifically to 3′-untranslated regions of multiple targets [12] and participate in cell growth, differentiation, metabolism, apoptosis, and other biological processes.

Mice with a miR-146a deficiency develop severe gouty arthritis as a result of upregulation of the expression of TRAK6, IRAK-1 and NALP3 inflammasome, thus increasing the degree of the inflammatory response [13]. miR-302b regulates the transcription of IL-1*β* and reduces the production of IL-1*β* by targeting IRAK4 and EphA2. Therefore, miR-302b can also be considered as a potential therapeutic target for gouty arthritis [13]. miR-488 and miR-920 have also been demonstrated to be reduced significantly in gouty arthritis patients, possibly via the targeting of 3′-UTR of IL-1*β* [14]. Overexpression of miR-155 in synovial monocytes of patients with acute gouty arthritis reduced the level of SHIP-1, activated the Akt/NF-kB pathway, and promoted the production of proinflammatory cytokines such as TNF-*α*, and IL-1*β* [15]. The Chuanhutongfeng mixture contains the herbs* Dioscorea nipponica Makino* (*Dioscoreaceae*),* Fallopia japonica* (*Polygonaceae*),* Lonicera japonica* (Caprifoliaceae),* Saposhnikovia divaricata* (Apiaceae),* Clematis chinensis* (*Ranunculaceae*),* Smilax glabra* (*Smilacaceae*),* Cyathula officinalis* (*Amaranthaceae*),* Ligusticum chuanxiong* (*Apiaceae*),* Dioscorea futschauensis* (*Dioscoreaceae*),* Pseudocydonia sinensis* (*Rosaceae*), and* Glycyrrhiza uralensis* (*Fabaceae*) as well as sodium alginate. The formula has been shown to play an anti-inflammatory role by inhibiting inflammation and infiltration in local synovial tissue, the production of white blood cells, and the conversion of inactive interleukin (IL)-1 to active IL-1*β* [16]. Chuanhutongfeng mixture can relieve the symptoms of CGA, and its clinical efficacy in the treatment of CGA was not inferior to colchicine but notably had a higher safety factor [17].

The aims of the present study were to screen differentially expressed miRNAs in CGA patients and healthy controls using miRNA microarray assay technology and to verify differentially expressed miRNAs via RT-qPCR. At the same time, we studied the effects of the Chuanhutongfeng mixture in the treatment of CGA and on differentially expressed miRNAs, which might have a regulatory role in CGA.

## 2. Materials and Methods

### 2.1. Patients

From May 2014 to February 2015, 255 patients with CGA in gout clinics of the Affiliated Hospital of Qingdao University and 30 age and gender-matched healthy subjects were selected for the trial. The fasting plasma of all participants was collected. The study protocol was approved by the Research Ethics Committee of the Affiliated Hospital of Qingdao University, and all patients provided written informed consent.

*Patient Inclusion Criteria.* (1) aged 18 to 70 years with no restriction on gender; (2) CGA, blood uric acid levels of males > 420 *μ*mol/L and females > 360 *μ*mol/L and not taking colchicine, nonsteroidal anti-inflammatory drugs, glucocorticoids, or other drugs; (3) not taking allopurinol, benzbromarone, thiazide diuretics, aspirin, pyrazinamide, nifedipine or other drugs that may have affected uric acid metabolism during the previous 3 months; (4) free of diseases that affect uric acid metabolism such as chronic renal dysfunction; (5) not having taken lipid-regulating drugs, angiotensin converting enzyme inhibitor (ACEI), or angiotensin receptor blockers (ARB) during the previous 3 months. 

*Patient Exclusion Criteria.* (1) allergic or hypersensitive to the test drug; (2) active liver disease or cirrhosis, or those whose serum alanine aminotransferase and glutamate transaminase exceeded the upper limit of the normal range by 1.5 times; (3) gastrointestinal ulcers; (4) Abnormal thyroid function; (5) blood creatinine levels greater than the upper limit of the reference range; (6) severe heart disease or a history of myocardial infarction in the previous 12 months; (7) taking xanthine; (8) rheumatoid arthritis; (9) using azathioprine, 6-mercaptopurine, theophylline, cyclophosphamide, trimethoprim, cyclosporine, thiazide diuretics, aspirin or other salicylic acid drugs, or losartan, or who received colchicine by intravenous administration; (10) secondary hyperuricemia caused by nephropathy, hematopathy or tumor radiotherapy and chemotherapy; (11) patients with brain diseases, abnormal judgment, and mental disorders; (12) alcohol or drug addiction; (13) pregnant, lactating or intending to get pregnant; (14) participating in other clinical trials within 3 months prior to screening for this trial; (15) active tuberculosis or malignant tumors; (16) complications due to other diseases; (17) taking glucocorticoid; (18) for any other reason the researcher did not consider it appropriate for an individual to participate.

### 2.2. miRNA Expression Profile in Preliminary Experiments

**Figure 1:**
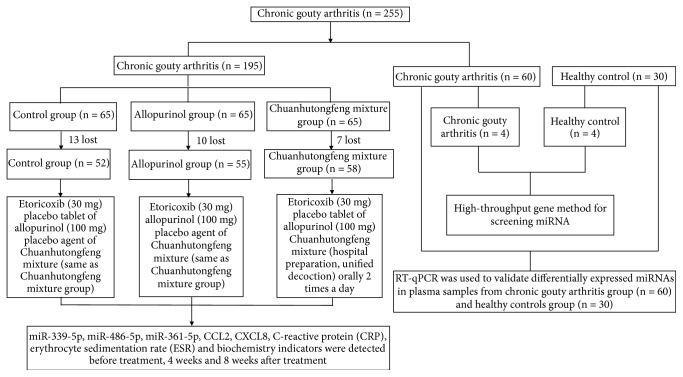
Flowchart of the study. CCL2: chemokine (C-C motif) ligand 2; CXCL8: chemokine (C-X-C motif) ligand 8; CRP: C-reactive protein; ESR: erythrocyte sedimentation rate.

The expression of miRNAs in plasma from eight subjects was screened by miRNA chip technology. There were four patients with CGA (3 males, 1 female: 50.20±8.90 years) and four healthy controls (3 males, 1 female: 49.70±8.70 years). There was no significant difference in age and gender between the 2 groups (Figure 1).

After obtaining expression profiles of plasma miRNAs, RT-qPCR was used to validate further the differentially expressed miRNAs in the CGA (60 patients) and control (30 healthy subjects) groups. There were 60 patients with CGA including 56 males and 4 females (50.80±9.00 years) and 30 healthy controls including 28 males and 2 females (50.00±8.90 years). There was no significant difference in age and gender between the two groups (Table 1).

### 2.3. microRNA Array Analysis Method (7th miRCURY*™* LNA Array: Ver. 18.0)

Total RNA of plasma samples was isolated using Trizol LS (Invitrogen, Canada) and purified with an RNeasy mini kit (Qiagen, Germany), and the quantity of RNA was determined using a NanoDrop 8000 spectrophotometer (Thermo Fisher Scientific, US). A miRCURY™ Array Power Labeling kit (Exiqon, Denmark) was used for microRNA labeling, and then Hy3™-labeled samples were hybridized on a miRCURY™ LNA Array (Exiqon). Chips were scanned using an Axon GenePix 4000B microarray scanner (Axon Instruments, CA, US) and the images were imported into GenePix Pro 6.0 software (Axon Instruments, CA, US) for grid alignment and data extraction. The replicated microRNAs were averaged and those with intensities ≥ 30 in all samples were selected for normalization. Differentially expressed microRNAs were identified by a volcano plot and a heat map (MEV ver. 4.8, TIGR).

### 2.4. Real-Time Quantitative PCR

To establish the accuracy of the microRNA assays, we used RT-qPCR to validate differentially expressed miRNAs in the plasma samples. Total RNA was isolated using Trizol LS (Invitrogen, Canada) and purified with an RNeasy mini kit (Qiagen, Germany), and the quantity of RNA was determined using a NanoDrop 8000 spectrophotometer (*vide supra*). RNA was reverse transcribed into cDNA using a Gene Amp PCR System 9700 (Applied Biosystems, US) and quantitative real-time PCR was performed with a ViiA 7 Real-time PCR System (Applied Biosystems, US). We used U6 as the internal reference to normalize the samples and relative expression levels of miRNAs were determined by the (2-△△ Ct) method. The primers used for RT-qPCR were designed using Primer 5.0 software (Supplementary Table 1).

### 2.5. Conceptual Design, Sample Size Estimation and Groupings

The design was a 1:1:1 prospective, randomized, double blind, double simulation, and parallel controlled study. It was estimated that ≥ 50 cases would be needed for each group, the estimated rate of abscission was about 20%, and 195 cases were included. Nomenclature has been used according to previous literature [18].

### 2.6. Random and Blinding Method

First, we selected the appropriate segment length and then determined the number of seeds according to Proc Plan in SAS statistical software, resulting in a random arrangement of 195 patients in the group.

The principle of double blinding was carried out including the generation of random numbers, the number of experimental drugs, the specific grouping of research subjects, the recording, management, and statistical analysis of data.

### 2.7. Medication

Chuanhutongfeng mixture is an improvement of the Tongfeng mixture which we developed before and contains* Rhizome dioscorea nipponica* (15g),* Rhizoma polygoni cuspidati *(15 g),* Caulis lonicerae* (30 g),* Radix saposhnikoviae* (15 g),* Radix clematidis *(15 g),* Rhizoma smilacis glabrae* (15 g),* Radix cyathulae* (15 g),* Rhizoma ligustici chuanxiong* (15 g),* Rhizoma dioscoreae hypoglaucae* (12 g),* Fructus chaenomelis* (15 g),* Radix glycyrrhizae* (6 g), and sodium alginate (1 g). First, the herbs were decocted twice (the first time for 2 h and the second time for 1 h), with water in a ratio of 1:10-20. Then the two decoctions were mixed, filtered, and then concentrated to 1,000 mL. Finally, the decoction was sterilized to obtain the Chuanhutongfeng mixture with a concentration of 169 g/1,000 mL. The mass spectrogram of the Chuanhutongfeng mixture showed that it contained resveratrol, chlorogenic acid, and loganin (Supplementary Figure 1). In the present study, the dosage for the treatment of chronic gouty arthritis comprised 125 mL Chuanhutongfeng mixture orally, twice a day.

The control group was given 30 mg etoricoxib orally, once a day, a placebo tablet of allopurinol, and a placebo agent of the Chuanhutongfeng mixture for a total course of treatment of 8 weeks. The Chuanhutongfeng mixture group was based on the control group plus Chuanhutongfeng mixture (hospital preparation, unified decoction) orally 2 times a day and a placebo tablet of allopurinol for a total course of treatment of 8 weeks. The allopurinol group was based on the control group plus allopurinol 100 mg administered orally 3 times a day and a placebo agent of the Chuanhutongfeng mixture.

### 2.8. Laboratory Measurements

Height, weight, blood pressure, and related biochemical indicators were measured before 4 and 8 weeks after treatment for all subjects. FPG (after overnight fasting for 10 h) was measured using the glucose oxidase method; serum ALT, AST, creatinine, serum uric acid, and blood lipids, including TG and TC, were measured using an automated biochemical analyzer (7600-020; Hitachi, Tokyo, Japan). Serum CRP levels were determined using a particle-enhanced immunoturbidimetric assay (Beckman Coulter Inc., Kraemer Blvd., CA, US).

### 2.9. Clinical Observation Descriptive Indices and Evaluation Scores

The curative effects including joint pain, swelling, and restricted movement scores were used in a range of scores, with a higher score reflecting the severity of the condition according to the following criteria after 4 weeks and 8 weeks of treatment.

#### 2.9.1. Joint Pain Score

The score was assessed by a numerical rating scale (NRS): no pain: 0 points; mild pain, tolerable, being able to live and sleep normally: 1-3 points; moderate pain, appropriate to affect sleep but tolerable: 4-6 points; severe pain with persistent pain, affecting sleep, requiring painkillers, or being associated with other symptoms or compulsive position: 7-10 points.

#### 2.9.2. Joint Swelling Score

There is no significant change in skin texture and apophysis, without joint effusion: 0 points; skin texture becoming lighter, and the apophysis being clearly visible with a small amount of joint effusion: 1 point; the skin texture basically disappearing, the apophysis being flat with the swollen skin, and the signs of the apophysis being not obvious with medium joint effusion: 2 points; the skin texture completely disappearing, the swelling skin being higher than the apophysis, the signs of apophysis disappearing, and the joint function and activity being limited with a mass joint effusion: 3 points.

#### 2.9.3. Restricted Movement Score

Joint movement was not restricted: 0 points; the movement was slightly restricted and could be engaged in normal activities: 1 point; restricted movement was obvious and not being able to engage in normal activities but being able to still take care of themselves: 2 points; the movements were completely limited, and patients could not take care of themselves on a daily basis: 3 points.

#### 2.9.4. Efficacy Measurements

According to the related CGA efficacy criteria utilized by Meng [19] and Zhao [20], this included the following: (1)* clinical cure*: each score of joint pain, swelling, and restricted movement being 0 to 1 point; (2)* markedly effective*: the decrease of the sum of 3 scores after treatment ≥ 2/3; (3)* effective*: between markedly effective and ineffective; (4)* ineffective*: the decrease of the sum of 3 scores after treatment ≤ 1/3.

#### 2.9.5. Primary Outcome Measures

This included the following: the correlation between clinical effects of CGA and expression levels of miR-339-5p, miR-486-5p, and miR-361-5p in the plasma of CGA patients before and after treatment.

#### 2.9.6. Secondary Outcome Measures

This included the following: the levels of CCL2, CXCL8, erythrocyte sedimentation rate (ESR), and C-reactive protein (CRP) in the plasma before and after the treatment.

### 2.10. Adverse Events (AE) and Drug Related Adverse Events

The intention-to-treat (ITT) analysis and per-protocol treated (PP) analysis were used in AE analyses. All cases lost in the Chuanhutongfeng mixture, allopurinol, and control groups were considered to be acute CGA attacks.

### 2.11. Statistical Analysis

SPSS ver. 16.0 statistical software (SPSS Inc., Chicago, IL, US) was used for data analysis. Normally distributed data are expressed as the $\overset{-}{x}\pm s$ and a Student's* t*-test was used to compare the differences between 2 groups; one-way analysis of variance (ANOVA) was used to compare differences among multigroups and the Bonferroni method was utilized for 2 groups after multiple comparisons and the chi-squared test for count data. Pearson linear correlation analysis was carried out to examine the relationship among variables. Comparison of the observational levels at different time points was performed using repeated measures analysis of variance and a general linear model was used to analyze the interactions of time, groups, and various indicators. The recurrence rate of CGA after treatment between the 3 groups was compared using intention-to-treat (ITT) analysis and the per-protocol treated (PP) analysis. Statistical significance was defined as* P *< 0.05.

## 3. Results

### 3.1. miRNA Screening

#### 3.1.1. Characteristics of Patients Used for miRNA Screening in the CGA and Healthy Groups

There were no significant differences in the baseline data and biochemical indexes compared to the healthy group, but SUA, ESR, and CRP were significantly elevated and there were gouty arthritis symptoms such as pain, swelling, and restricted movement scores in the CGA group (Table 1). Thus, we selected four cases for screening miRNA gene profiles in each of the two groups.

#### 3.1.2. Volcano Plot and Heat Map Results from microRNA Array Analysis of the Two Groups of Patients

Heatmap (Supplementary Figure 2) and volcano plot (Supplementary Figure 3) obtained after scanning by using Exqion's LNA™ microRNA Microarray technology revealed a dendrogram of differentially expressed miRNAs and significantly different expression profiles of miRNAs in the plasma obtained from the CGA and healthy groups (*P <* 0.05).

#### 3.1.3. Abnormally Expressed miRNAs Screened between the CGA and Healthy Groups

There were 48 miRNAs with higher or lower expression levels in the plasma of patients with CGA, of which 36 were significantly upregulated ≥ 1.5 times compared to the control group. miRNAs which were upregulated or downregulated > 2 times are shown in Supplementary Table 2.

#### 3.1.4. Signaling Pathways Related to Differentially Expressed miRNAs in the CGA and Healthy Control Groups

Based on GO analysis and pathway analysis, the pathways of the target genes that may be regulated by differentially expressed microRNAs were analyzed (Supplementary Figure 4). The first 10 possible biological pathways of target genes and upregulated and downregulated microRNAs in the CGA group are reported. The ordinate was the signal pathway or biological process and the abscissa was the enrichment score, which was calculated from -log10 (*P*-value). The larger the score the smaller the* P*-value, indicating that the signal pathway was more significant. Therefore, the most likely pathways involved in the upregulation of microRNAs were glycosphingolipid biosynthesis, pertussis, and the neurotrophin-signaling pathways. The pathways involved in downregulation of miRNAs may be the mRNA surveillance pathway, hedgehog signaling pathway, and legionellosis. The miRNAs with altered expression were further analyzed for levels of miRNAs (miR-339-5p, miR-486-5p, and miR-361-5p) that may have been associated with CGA.

#### 3.1.5. miRNAs Related with CGA Pathogenesis Were Selected between the Two Groups

According to the literature and our analysis of expression differences, we chose three miRNAs that may play important roles in the pathogenesis of CGA (miR-339-5p, miR-486-5p, and miR-361-5p). RT-qPCR was used to detect microRNA expression in plasma samples obtained from 60 patients with CGA and 30 healthy controls. The relative expression levels of miR-339-5p, miR-486-5p, and miR-361-5p in the CGA group of patients were significantly higher than in the healthy control group (4.28-fold, 7.02-fold, and 5.83-fold, respectively) (Table 2).

### 3.2. Effects of Different Medication on Clinical Improvement and Occurrences of Gout Specific miRNA Patterns

From May 2014 to February 2015, there were 255 cases of CGA in the gout clinic of the Affiliated Hospital of Qingdao University, and finally 195 cases were enrolled. There were 186 males and 9 females, with an average age of 50.50±9.30 years. In the allopurinol group, there were a total of 55 cases comprised of 53 males and 2 females, with an average age of 50.50±8.90 years. In the Chuanhutongfeng mixture group, there were 58 patients in the Chuanhutongfeng mixture group comprised of 55 males and 3 females, with an average age of (51.10±9.10) years. Finally there were 52 patients in the control group, comprised of 50 males and 2 females with an average age of 49.90±9.00 years.

All patients have been in treatments before the trial. There were no statistical differences in age, gender, miR-339-5p, miR-486-5p, miR-361-5p, blood pressure, FPG, blood lipids, serum uric acid, body mass index, eGFR, ALT, AST, CCL2, CXCL8, CRP, pain scores, swelling scores, and restricted movement scores between the three groups (Table 3).

Improvement in the clinical symptoms of the three groups of patients after 4/8 weeks of treatment is shown in Table 4. After 4 weeks of treatment, the cure rates of the Chuanhutongfeng mixture and allopurinol groups were 37.93% and 32.73%, respectively (compared with the control group,* P *< 0.05). After 8 weeks of treatment, the cure rates of the Chuanhutongfeng mixture and allopurinol groups were 72.41% and 49.09%, which were significantly greater than that of the control group (*P *< 0.05). In addition, the cure rate was higher in the Chuanhutongfeng mixture group than in the allopurinol group (*P *< 0.05).

#### 3.2.1. Comparison of Observation Indexes after Treatment of the Three Groups at Different Follow-Up Durations

SUA values were significantly more reduced after 4 weeks and 8 weeks of treatment in the Chuanhutongfeng mixture and the allopurinol groups compared to the control, but the SUA reduction in the Chuanhutongfeng mixture patients was significantly greater than in the allopurinol group after 8 weeks of treatment.

*After 4 week treatments*. eGFR was significantly enhanced in the Chuanhutongfeng mixture patients compared to the allopurinol and control treated patients, which showed that the Chuanhutongfeng mixture functioned to protect the kidneys. After 4 weeks of treatment, pain, swelling and restricted movement scores, and ESR, CRP, SUA, and CCL2 levels were significantly decreased in the allopurinol and Chuanhutongfeng mixture groups compared to the control (*P *< 0.05). The CXCL8 and CCL2 levels were significantly lower than before treatment in both the Chuanhutongfeng mixture and the allopurinol groups compared to the controls. Only miR-486-5p was significantly enhanced after 4 weeks allopurinol treatment, but miR-486-5p, miR-361-5p, and miR-339-5p levels in the Chuanhutongfeng mixture group were all significantly elevated after 4 weeks of medication compared to the control (*P *< 0.05).

*After 8 week treatments.* After 8 weeks of treatment, joint pain, swelling and restricted movement scores, ESR, CRP, and the plasma CCL2 and CXCL8 level were significantly decreased in both Chuanhutongfeng mixture and allopurinol treated patients compared to the controls (*P *< 0.05), but CCL2 and CXCL8 serum concentrations were significantly more reduced in the Chuanhutongfeng mixture group compared to the allopurinol group. In comparison to the controls, plasma levels of miR-486-5p, miR-339-5p, and miR-361-5p were upregulated (*P *< 0.05) in both the Chuanhutongfeng mixture and the allopurinol groups, but the elevation was significantly greater in the Chuanhutongfeng mixture group compared to allopurinol treated patients (*P *< 0.05) (Table 5).

#### 3.2.2. Analysis of Correlations between Levels of Plasma miR-486-5p and miR-361-5p, Pain Scores, and CRP in CGA Patients

**Figure 2:**
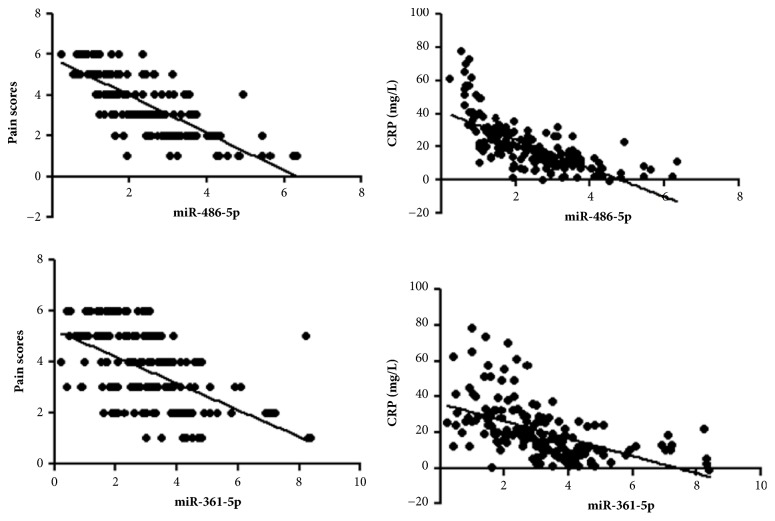
Analysis of the correlation between levels of plasma miR-486-5p and miR-361-5p, pain scores and CRP in CGA patients. CRP: C-reactive protein; CGA: chronic gouty arthritis.

The linear correlation analysis of levels of plasma miR-486-5p and miR-361-5p with pain scores and CRP reflecting the patients' condition are shown in Figure 2. The plasma miR-486-5p and miR-361-5p levels were negatively correlated with the pain scores (*r *= -0.761, -0.576, and* P *< 0.05) and CRP (*r *= -0.697, -0.529, and* P *< 0.05).

#### 3.2.3. Analysis of Acute CGA Exacerbation Rates after 8 Weeks of Treatment in the Three Groups

There was one case (1.72%) of an acute CGA attack in the Chuanhutongfeng mixture group, three cases (5.45%) of recurrence in the allopurinol group, and six cases (11.54%) of recurrence in the control group after 8 weeks of treatment in a PP-analysis. An ITT-analysis revealed five acute CGA attack cases (7.68%) in the Chuanhutongfeng mixture group, eight cases (12.31%) of recurrences in the allopurinol group and eleven cases (16.92%) in the control group. However, both analyses did not show significant differences between the groups (Table 6).

#### 3.2.4. Adverse Reactions in the Three Groups

All symptoms of adverse effects including diarrhea occurred in the control group during the course of the treatment. In the allopurinol group abnormal liver functions with elevated ALT, AST, and bilirubin levels were noted and an inability to tolerate the bitter taste of Chinese medicine and the associated mild diarrhea occurred in one patient of the Chuanhutongfeng group.

## 4. Discussion

We screened gout-specific miRNAs using microarray chips and selected miR-486-5p, miR-339-5p, and miR-361-5p that were differentially expressed in patients with CGA, after which we performed a large sample of RT-qPCR verification. The results showed that the expression levels of miR-486-5p, miR-339-5p, and miR-361-5p in plasma of patients with CGA were significantly lower than in healthy controls. Interestingly, the downregulation of the same three miRNAs in rotator cuff tendinopathy patients indicated concomitant glenohumeral arthritis [21]. The plasma miR-486-5p, miR-361-5p, and miR-339-5p levels were increased after 8 weeks of treatment with the Chuanhutongfeng mixture and allopurinol compared with the control (etoricoxib only) group and upregulation of plasma miR-486-3p and miR-361-5p in the Chuanhutongfeng mixture group was significantly greater than in the allopurinol group. Simultaneously, the Chuanhutongfeng mixture and allopurinol treatment effectively reduced the levels of blood uric acid. The expressions of the inflammatory cytokines CCL2 and CXCL8 were also significantly reduced after 8 weeks of treatment in the Chuanhutongfeng mixture and allopurinol groups compared to the controls. The Chuanhutongfeng mixture produced fewer side effects compared with allopurinol indicating that the Chuanhutongfeng mixture may have better clinical efficacy than allopurinol in preventing an acute attack of CGA.

According to previous research, miR-339-5p inhibited the expressions of the proinflammatory factors IL-6, IL-1*β*, and TNF-*α* by abolishing NF-*κ*B activity [22] and miR-361-5p is downregulated in interferon-positive antiphospholipid syndrome and systemic lupus erythematosus dendritic cells [23]. In addition miR-486-5p has been shown to inhibit pulmonary fibrosis [24]. There are no publications on the involvement of these miRNAs in gouty arthritis or correlations with CCL2 and CXCL8. However, CXCL8 is transcribed by NF-*κ*B and its expression is stimulated by TNF-*α*, which are targets of miR-339-5p [25]. Since CCL2 has been shown to be upregulated by NF-*κ*B and TNF-*α* [26, 27], both downregulations might be attributed to miR-339-5p activity. The roles of miR-486-5p and miR-361-5p in CCL2 and CXCL8 expressions are somewhat unclear, but according to the miRBase database (http://www.mirbase.org/) there are 151 predicted target genes for hsa-miR-486-5p and 340 predicted target genes for hsa-miR-361-5p, from which some might be involved indirectly in CCL2 or CXCL8 expressions.

The main active component of Chuanhutongfeng mixture is resveratrol, and the existing literature has reported that resveratrol can significantly reduce the inflammatory and apoptotic damage of LPS-induced cells by upregulating miR-146b, thereby inactivating the NF-*κ*B and p38MAPK pathways [28]. The results of animal experiments also showed that resveratrol effectively inhibited inflammation [29, 30]. Resveratrol can also reduce endothelial inflammation by reducing ICAM-1 expression and mediate protection through the miR-221/222/AMPK/p38/NF-*κ*B pathway [31]. In a previous study, it was shown that resveratrol could effectively treat acute gout arthritis [32]. This agent can significantly reduce joint synovial tissue edema and inflammatory cell infiltration and improve synovial hyperplasia, and its efficacy may be achieved by inhibiting the local aggregation of white blood cells into the inflammatory region and secretion of IL-1*β* [33].

We will continue to examine whether resveratrol, the main component of Chuanhutongfeng mixture, can treat CGA by modulating the expression levels of miR-339-5p, miR-486-5p, and miR-361-5p, thus inhibiting the expression of inflammatory factors. In contrast to differential miR-339-5p, miR-486-5p, and miR-361-5p expression pattern, the roles of CCL2 and CXCL8 in gout patients are well known [34–37].

However, due to the small number of cases in our study, we need further validation with well-designed, large cohort clinical trials. The downregulation of miR-486-5p, miR-339-5p, and miR-361-5p in plasma may play a role in the pathogenesis of CGA, and upregulation of the expression of miR-486-5p, miR-339-5p, and miR-361-5p may be the mechanism of action(s) of the Chuanhutongfeng mixture and allopurinol in the treatment of CGA.

There are a number of limitations to our research findings. First, the sample size was small and the follow-up time was brief. Second, the mechanism of upregulation of miR-339-5p, miR-486-5p, and miR-361-5p by the Chuanhutongfeng mixture is not fully understood and a direct association of miR-339-5p, miR-486-5p, and miR-361-5p with CCL2 and CXCL8 could not be established. It will be necessary to design a new large cohort clinical study and carry out more in-depth basic research to clarify our findings.

## 5. Conclusions

The expression levels of miR-339-5p, miR-486-5p, and miR-361-5p may be associated with CGA. Chuanhutongfeng mixture can significantly upregulate the expression of miR-339-5p, miR-486-5p, and miR-361-5p and reduce the levels of CCL2 and CXCL8. Its clinical efficacy is similar to allopurinol, but with significantly lower side effects. The expression levels of miR-486-5p and miR-361-5p in plasma were correlated with joint pain scores and CRP in patients with CGA and may be used as biological indicators to determine the condition of CGA patients and the likely clinical outcomes.

## Figures and Tables

**Table 1:** Baseline data for high-throughput gene screening and RT-qPCR validation in the healthy control and CGA groups.

Indicators	Healthy control group (n = 30)	CGA group (n = 60)	*P*-value
Age	50.00 ± 8.90	50.80 ± 9.00	0.691
Gender (% of males)	28 (93.33)	56 (93.33)	1.000
BMI (kg/m^2^)	25.20 ± 1.10	25.50 ± 1.30	0.281
Systolic pressure (mmHg)	132.00 ± 8.00	133.00 ± 7.00	0.544
Diastolic pressure (mmHg)	76.00 ± 6.00	77.00 ± 6.00	0.458
FBG (mmol/L)	5.10 ± 0.60	5.20 ± 0.50	0.406
TC (mmol/L)	4.90 ± 0.50	5.10 ± 0.50	0.077
TG (mmol/L)	2.10 ± 0.80	2.20 ± 0.90	0.608
ALT (U/L)	30.00 ± 9.00	31.10 ± 8.50	0.572
AST (U/L)	25.20 ± 7.80	26.80 ± 8.30	0.382
eGFR(ml/min/1.73m^2^)	111.00 ± 6.50	110.00 ± 7.50	0.392
SUA (*μ*mol/L)	330.00 ± 70.00	529.00 ± 76.00	< 0.001
ESR (mm/h)	10.00 ± 5.50	33.00 ± 15.70	< 0.001
CRP (mg/L)	3.10 ± 2.20	22.20 ± 11.70	< 0.001
Pain scores	0	3.60 ± 0.80	-
Swelling scores	0	1.90 ± 0.89	-
Restricted movement scores	0	1.90 ± 0.90	-

CGA: chronic gouty arthritis; BMI: body mass index; FBG: Fasting blood-glucose; TC: total cholesterol; TG: triglyceride; ALT: alanine aminotransferase; AST: aspartate aminotransferase; eGFR: estimate glomerular filtration rate; SUA: serum uric acid; ESR: erythrocyte sedimentation rate; CRP: C-reactive protein.

**Table 2:** The relative expression of 3 miRNAs in plasma in the CGA and healthy control groups.

	Healthy control group (n = 30)	CGA group (n = 60)	*P*-value
miR-486-5p	17.12 ± 2.33	2.44 ± 1.26	< 0.001
miR-339-5p	20.44 ± 2.89	4.79 ± 2.13	< 0.001
miR-361-5p	18.18 ± 3.23	3.12 ± 1.44	< 0.001

CGA: chronic gouty arthritis.

**Table 3:** Baseline information of patients in the 3 groups.

Indicators	Control group (n = 52)	Allopurinol group (n = 55)	Chuanhutongfeng mixture group (n = 58)	*P*-value
Age	49.90 ± 9.00	50.50 ± 8.90	51.10 ± 9.10	0.784
Gender (% of males)	50 (96.15)	53 (96.36)	55 (94.83)	0.908
BMI (kg/m^2^)	25.33 ± 1.12	25.46 ± 1.06	25.50 ± 1.26	0.726
Systolic pressure (mmHg)	133.00 ± 7.00	132.00 ± 8.00	134.00 ± 7.00	0.354
Diastolic pressure (mmHg)	76.00 ± 5.00	75.00 ± 7.00	75.00 ± 6.00	0.618
Disease duration (years)	9.90 ± 5.11	9.91 ± 5.42	9.87 ± 5.50	0.999
Tophus (n, %)	26 (50.00)	28 (50.91)	30 (51.72)	0.984
FBG (mmol/L)	5.12 ± 0.58	5.21 ± 0.46	5.17 ± 0.63	0.710
TC (mmol/L)	4.89 ±0.45	5.03 ± 0.46	5.10 ± 0.47	0.057
TG (mmol/L)	2.17 ± 0.85	2.21 ± 0.72	2.16 ± 0.81	0.940
ALT (U/L)	30.20 ± 9.50	29.50 ± 8.80	31.70 ± 8.40	0.406
AST (U/L)	25.10 ± 7.70	24.80 ± 7.40	27.90 ± 8.50	0.074
eGFR(ml/min/1.73m^2^)	110.10 ± 7.40	111.50 ± 7.50	110.40 ± 7.70	0.339
SUA (*μ*mol/L)	532.00 ± 69.00	529.00 ± 72.00	530.00 ± 74.00	0.976
ESR (mm/h)	31.00 ± 14.20	33.90 ± 15.70	32.20 ± 16.30	0.622
CRP (mg/L)	21.30 ± 9.20	20.40 ± 10.80	22.00 ± 12.10	0.734
Pain score	3.54 ± 1.57	3.71 ±1.58	3.64 ±1.38	0.843
Swelling score	1.97 ± 0.89	1.97 ± 0.95	1.97 ± 0.88	1.000
Restricted movement score	1.98 ± 0.72	1.98 ± 0.89	1.99± 0.88	0.997
CCL2 (pg/mL)	538.00 ± 223.00	545.00 ± 175.00	572.00 ± 160.00	0.597
CXCL8 (pg/mL)	619.00 ± 199.00	643.00 ± 181.00	645.00 ± 218.00	0.757
miR-486-5p	2.51 ± 1.22	2.31 ± 1.19	2.24 ± 1.31	0.505
miR-339-5p	4.90 ± 1.98	4.93 ± 2.16	4.64 ± 2.18	0.725
miR-361-5p	3.27 ± 1.80	3.15 ± 1.77	2.98 ± 1.47	0.661

BMI: body mass index; FBG: Fasting blood-glucose; TC: Total cholesterol; TG: triglyceride; ALT: alanine aminotransferase; AST: aspartate aminotransferase; eGFR: estimate glomerular filtration rate; SUA: serum uric acid; ESR: erythrocyte sedimentation rate; CRP: C-reactive protein; CCL2: chemokine (C-C motif) ligand 2; CXCL8: chemokine (C-X-C motif) ligand 8.

**Table 4:** Comparison of clinical efficacy in the 3 groups after 4 and 8 weeks treatment.

Clinical efficacy index	Control group (n = 52)		Allopurinol group (n = 55)		Chuanhutongfeng mixture group (n = 58)	
	4 weeks	8 weeks	4 weeks	8 weeks	4 weeks	8 weeks
Cure	3 (5.77%)	4 (7.69%)	18 (32.73%)^a^	27 (49.09%)^b^	22 (37.93%)^a^	42 (72.41%)^bd^
Markedly effective	10 (19.23%)	12 (23.08%)	20 (36.36%)	15 (27.27%)	20 (34.48%)	12 (20.69%)
Effective	27 (51.92%)	25 (48.08%)	15 (27.27%)	11 (20.00%)	15 (25.86%)	4 (6.90%)
Ineffective	12 (23.08%)	11 (21.15%)	2 (3.64%)	2 (3.64%)	1 (1.72%)	0 (0.00%)

*Note.* Compared with the control group after 4 weeks ^a^*P *< 0.05, 8 weeks, ^b^*P *< 0.05; compared with the allopurinol group after 4 weeks, ^c^*P*< 0.05, 8 weeks, ^d^*P*< 0.05.

**Table 5:** Comparison of differences in observation indicators after 4 and 8 weeks treatment in the 3 groups.

Indicators	Control group (n = 52)		Allopurinol group (n = 55)		Chuanhutongfeng mixture group (n = 58)	
	4 weeks	8 weeks	4 weeks	8 weeks	4 weeks	8 weeks
∆ESR (mm/h)	-8.00 ± 3.80	-9.00 ± 3.60	-16.20 ± 9.30^a^	-17.30 ± 9.20${}^{\overset{\;}{b}}$	-16.40 ± 4.30^a^	-19.00 ± 4.60^b^
∆CRP (mg/L)	-1.20 ± 0. 50	-2.50 ± 0.50	-11.80 ± 7.80^a^	-12.00 ±7.70^b^	-15.40 ± 8.90^ac^	-15.50 ± 8.90^bd^
∆Pain score	-1.44 ± 0.51	-1.55 ± 0.50	-2.32 ± 0.60^a^	-2.41 ± 0.53^b^	-2.36 ± 0.53^a^	-2.56 ± 0.46^b^
∆Swelling score	-1.11 ± 0.42	-1.15 ± 0.42	-1.46 ± 0.51^a^	-1.47 ± 0.41^b^	-1.47 ± 0.47^a^	-1.51 ± 0.47^b^
∆Restricted movement score	-1.00 ± 0.49	-1.03 ± 0.49	-1.47 ± 0.48^a^	-1.48 ± 0.48^b^	-1.49 ± 0.50^a^	-1.53 ± 0.46^b^
∆SUA (*μ*mol/L)	-14.00 ± 12.00	-26.00 ± 23.00	-64.00 ± 21.00^a^	-70.00 ± 21.00^b^	-90.00 ± 47.00^a^	-170.00 ± 49.00^bd^
∆eGFR (ml/min/1.73 m^2^)	0.40 ± 2.10	0.40 ± 2.90	-3.00 ± 2.70^a^	-3.90 ± 3.60^b^	3.20 ± 1.70^ac^	5.70 ± 2.60^bd^
∆FPG (mmol/L)	-0.05± 0.22	-0.10 ± 0.22	-0.10 ± 0.25	-0.10 ± 0.25	-0.22 ± 0.32	-0.20 ± 0.32
∆TG (mmol/L)	-0.56 ± 0.37	-0.58 ± 0.37	-0.63 ± 0.36	-0.68 ± 0.35	-0.61 ± 0.31	-0.65 ± 0.31
∆TC (mmol/L)	-0.22 ± 0.23	-0.18 ± 0.23	-0.31 ± 0.22	-0.36 ± 0.22	-0.43 ± 0.19	-0.49± 0.19
∆CCL2 (pg/mL)	-21.00 ± 24.00	-32.00 ± 24.00	-88.00 ± 76.00^a^	-163.00 ± 136.00^b^	-97.00 ± 89.00^a^	-174.00 ± 129.00^bd^
∆CXCL8 (pg/mL)	-14.00 ± 18.00	-21.00 ± 18.00	-22.00 ± 28.00^a^	-43.00 ± 38.00^b^	-93.00 ± 93.00^ac^	-212.00 ± 133.00^bd^
∆miR-486-5p	0.12 ± 0.12	0.15 ± 0.16	0.58 ± 0.53^a^	3.28 ± 0.55^b^	0.78 ± 0.67^a^	5.78 ± 1.72^bd^
∆miR-339-5p	0.08 ± 0.16	0.11 ± 0.19	0.09 ± 0.14	1.69 ± 0.47^b^	0.60 ± 0.49^ac^	4.25 ± 1.61^bd^
∆miR-361-5p	0.13 ± 0.10	0.28± 0.15	0.25 ± 0.15	4.43 ± 1.06^b^	0.63 ± 0.20^a^	6.53 ± 2.79^bd^

*Note.* ESR: erythrocyte sedimentation rate; CRP: C-reactive protein; eGFR: estimate glomerular filtration rate; FBG: fasting blood-glucose; TG: triglyceride; TC: total cholesterol; SUA: serum uric acid; CCL2: chemokine (C-C motif) ligand 2; CXCL8: chemokine (C-X-C motif) ligand 8. ∆ values: the value of index which were measured before or after treatment subtracted their baseline value; compared with the control group after 4 weeks ^a^*P *< 0.05, 8 weeks, ^b^*P *< 0.05; compared with the allopurinol group after 4 weeks, ^c^*P*< 0.05, 8 weeks, ^d^*P*< 0.05.

**Table 6:** Comparison of acute CGA attack rates in the three groups during the study.

	Control group	Allopurinol group	Chuanhutongfeng mixture group	*P*-value
PP-analysis (cases/total, %)	6/52 (11.5%)	3/55 (5.45%)	1/58 (1.72%)	0.096
ITT-analysis (cases/total, %)	11/65 (16.92%)	8/65 (12.31%)	5/65 (7.69%)	0.277

CGA: chronic gouty arthritis; PP, per protocol; ITT, intention-to-treat.

## Data Availability

The data used to support the findings of this study are available from the corresponding author upon request.

## Supplementary Materials

Supplementary Materials
** Supplementary Table** **1**. The target sequences of miRNAs and U6.** Supplementary Table ****2**. Specific high- and low-expressed miRNAs in plasma of patients with chronic gout (upregulation or downregulation > 2 times).** Supplementary Figure ****1**. The mass spectrum of Chuanhutongfeng mixture. 1 chlorogenic acid: 205.6 *μ*g/mL; 2 loganin: 36.2 *μ*g/mL; 3 resveratrol: 149.2 *μ*g/mL.** Supplementary Figure ****2**. Heat map of differential miRNAs expression in the CGA and healthy groups. The left panel shows the clustering of miRNAs; the top panel shows clustering between samples; red indicates that the relatively high level expression is upregulated by more than 1.5 times, and green indicates the relatively low level expression is downregulated by more than 1.5 times (CG was the CGA group and C was the healthy group). CGA: chronic gouty arthritis.** Supplementary Figure ****3**. Volcano plot. The volcano plot can visually show the expression profile of differential miRNAs between the CGA group and the healthy group (CG vs C). The abscissa represents the ratio between the 2 groups, and the ordinate represents the* P*-value. Two vertical lines represent the ratio of upregulation and downregulation of 1.5 times, and the horizontal line represents* P* = 0.05. Therefore, the red points in the plot represent the differentially expressed miRNAs with statistical significance and a ratio of 1.5-fold difference. CGA: chronic gouty arthritis.** Supplementary Figure ****4**. (a) Upregulated miRNAs may participate in regulating signaling pathways; (b) Downregulated miRNAs may participate in regulating signaling pathways.
