# Supplementary material for: A Randomized Controlled Trial of Chuanhutongfeng Mixture for the Treatment of Chronic Gouty Arthritis by Regulating miRNAs
Source: Evid Based Complement Alternat Med. 2019 Feb 3;2019:5917269. doi: 10.1155/2019/5917269 (PMC6378063; doi:10.1155/2019/5917269)
Supplement: Supplementary Materials — Supplementary Table 1. The target sequences of miRNAs and U6. Supplementary Table 2. Specific high- and low-expressed miRNAs in plasma of patients with chronic gout (upregulation or downregulation > 2 times). Supplementary Figure 1. The mass spectrum of Chuanhutongfeng mixture. 1 chlorogenic acid: 205.6 μg/mL; 2 loganin: 36.2 μg/mL; 3 resveratrol: 149.2 μg/mL. Supplementary Figure 2. Heat map of differential miRNAs expression in the CGA and healthy groups. The left panel shows the clustering of miRNAs; the top panel shows clustering between samples; red indicates that the relatively high level expression is upregulated by more than 1.5 times, and green indicates the relatively low level expression is downregulated by more than 1.5 times (CG was the CGA group and C was the healthy group). CGA: chronic gouty arthritis. Supplementary Figure 3. Volcano plot. The volcano plot can visually show the expression profile of differential miRNAs between the CGA group and the healthy group (CG vs C). The abscissa represents the ratio between the 2 groups, and the ordinate represents the P-value. Two vertical lines represent the ratio of upregulation and downregulation of 1.5 times, and the horizontal line represents P = 0.05. Therefore, the red points in the plot represent the differentially expressed miRNAs with statistical significance and a ratio of 1.5-fold difference. CGA: chronic gouty arthritis. Supplementary Figure 4. (a) Upregulated miRNAs may participate in regulating signaling pathways; (b) Downregulated miRNAs may participate in regulating signaling pathways. [file 5917269.f1.docx]

**Supplementary Table 1.** The target sequences of miRNAs and U6

| miRNA | Target sequences |
| --- | --- |
| U6 | GSP:5’GCTTCGGCAGCACATATACTAAAAT3’  R:5’ GTGCAGGGTCCGAGGT3’ |
| hsa-miR-339-5p-RT | GSP:5’ cgctccctgtcctccag 3’  R:5’ GTGCAGGGTCCGAGGT3’ |
| hsa-miR-486-5p-RT | GSP:5’CCGTCCTGTACTGAGCTGC 3’  R:5’ GTGCAGGGTCCGAGGT3’ |
| hsa-miR-361-5p -RT | GSP:5’ CCGCTTATCAGAATCTCCAG 3’  R:5’ GTGCAGGGTCCGAGGT3’ |

**Supplementary Table 2.** Specific high- and low-expressed miRNAs in plasma of patients with chronic gout (upregulation or downregulation > 2 times)

| **miRNAs name** | **Fold changes** | ***P*-value** |
| --- | --- | --- |
| hsa-miR-4324 | 5.176 | 0.006 |
| hsa-miR-765 | 3.486 | 0.021 |
| hsa-miR-92a-2-5p | 3.467 | 0.009 |
| hsa-miR-4738-3p | 3.450 | 0.035 |
| hsa-miR-5190 | 3.445 | 0.032 |
| hsa-miR-631 | 3.418 | 0.047 |
| hsa-miR-4531 | 3.412 | 0.018 |
| hsa-miR-1471 | 3.360 | 0.005 |
| hsa-miR-302a-3p | 3.144 | 0.040 |
| hsa-miR-4251 | 3.071 | 0.009 |
| hsa-miR- B1-5p | 3.015 | 0.008 |
| hsa-miR-204-5p | 2.970 | 0.046 |
| hsa-miR-4634 | 2.777 | 0.012 |
| hsa-miR-874-3p | 2.632 | 0.046 |
| hsa-miR-4726-5p | 2.513 | 0.048 |
| hsa-miR- H1 | 2.487 | 0.014 |
| hsa-miR-502-5p | 2.327 | 0.020 |
| hsa-miR-639 | 2.259 | 0.015 |
| hsa-miR-5580-5p | 2.244 | 0.019 |
| hsa-miR-5004-3p | 2.208 | 0.018 |
| hsa-miR-660-3p | 2.170 | 0.024 |
| hsa-miR- H8-5p | 2.111 | 0.002 |
| hsa-miR-205-3p | 2.082 | 0.048 |
| hsa-miR-4632-3p | 2.034 | 0.012 |
| hsa-miR-4748 | 2.027 | 0.002 |
| hsa-miR-505-5p | 2.018 | 0.008 |
| hsa-miR-151a-3p | 0.268 | 0.016 |
| hsa-miR-326 | 0.314 | 0.034 |
| hsa-miR-361-5p | 0.354 | 0.027 |
| hsa-miR-339-5p | 0.317 | 0.017 |
| hsa-miR-423-3p | 0.353 | 0.038 |
| hsa-miR-486-5p | 0.393 | 0.048 |
| hsa-miR-584-5p | 0.357 | 0.032 |
| hsa-miR-4288 | 0.397 | 0.013 |
| hsa-miR-130b-3p | 0.446 | 0.019 |
| hsa-miR-548l | 0.475 | 0.016 |

Note: Fold changes > 1 indicates high expression and < 1 indicates low expression

**Supplementary Figures**

**
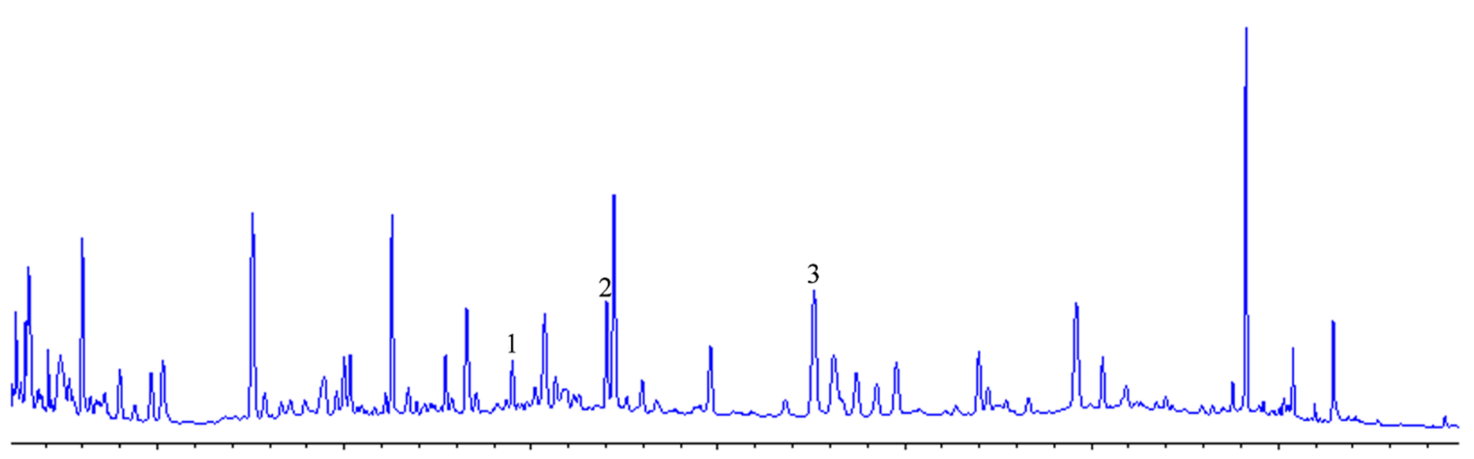
Supplementary Figure 1.** The mass spectrum of Chuanhutongfeng mixture. 1 chlorogenic acid: 205.6 µg/mL; 2 loganin: 36.2 μg/mL; 3 resveratrol: 149.2 μg/mL

**
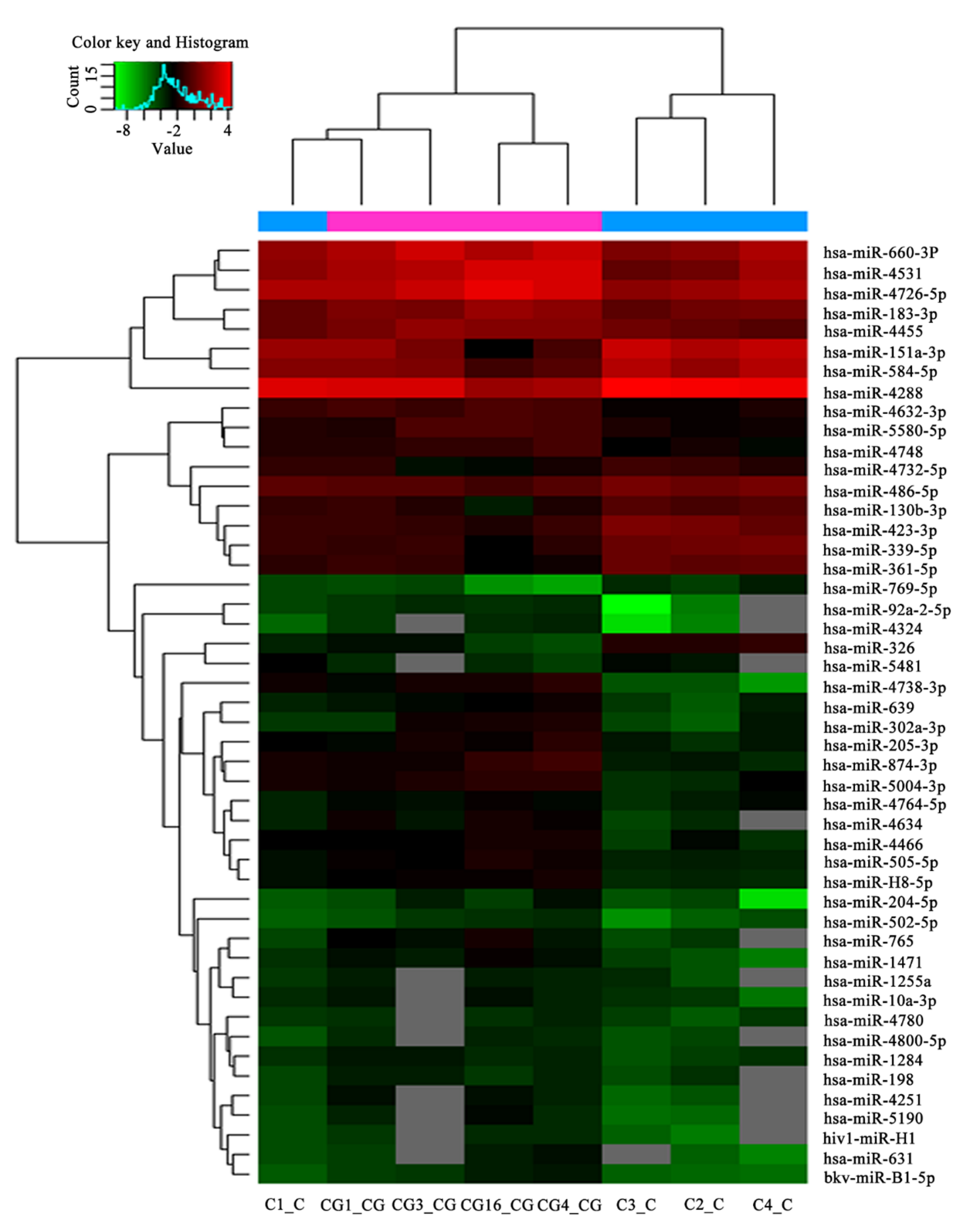
Supplementary Figure 2.** Heat map of differential miRNAs expression in the CGA and healthy groups. The left panel shows the clustering of miRNAs; the top panel shows clustering between samples; red indicates the relatively high level expression is upregulated by more than 1.5 times, and green indicates the relatively low level expression is downregulated by more than 1.5 times (CG was the CGA group and C was the healthy group).

CGA: chronic gouty arthritis

**
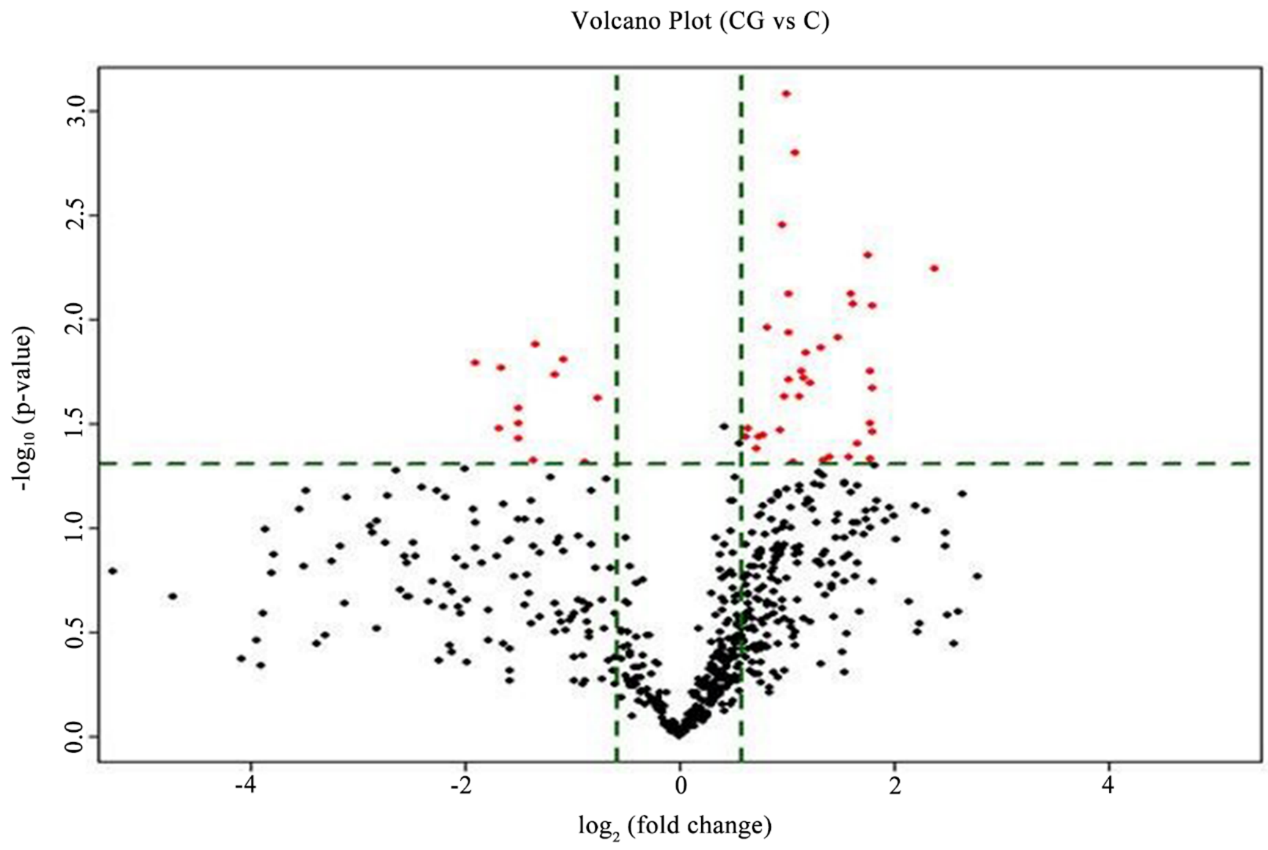
Supplementary Figure 3**. Volcano plot. The volcano plot can visually shows the expression profile of differential miRNAs between the CGA group and the healthy group (CG vs C). The abscissa represents the ratio between the 2 groups, and the ordinate represents the *P*-value. Two vertical lines represent the ratio of upregulation and downregulation of 1.5 times, and the horizontal line represents *P* = 0.05. Therefore, the red points in the plot represents the differentially expressed miRNAs with statistical significance and a ratio of 1.5-fold difference.

CGA: chronic gouty arthritis

**
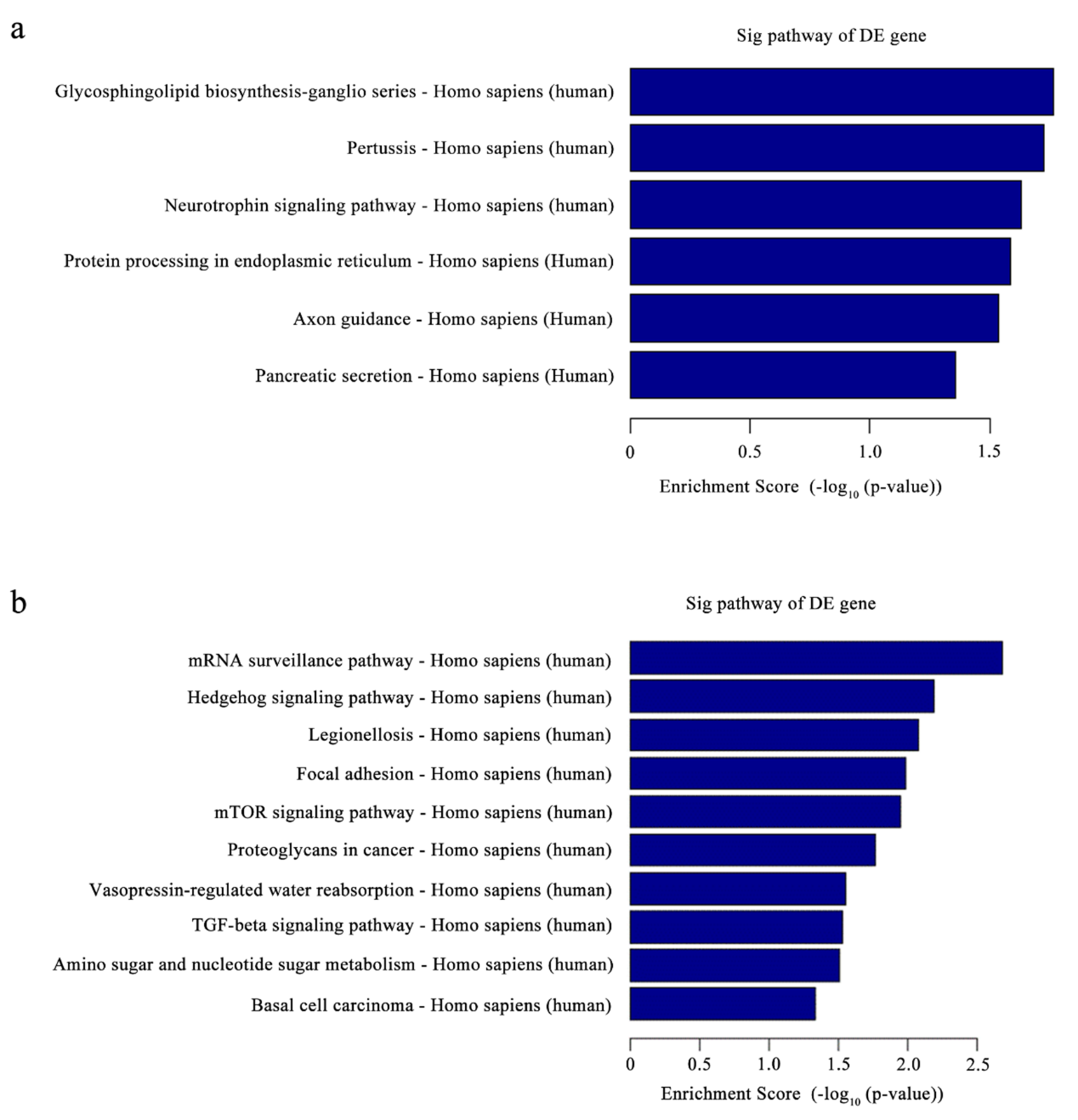
Supplementary Figure 4.** a) Upregulated miRNAs may participate in regulating signaling pathways; b) Downregulated miRNAs may participate in regulating signaling pathways.
